# Impact of the Bronx Community Health Leaders Program for Socioeconomically Disadvantaged Prehealth Students

**DOI:** 10.1089/heq.2021.0065

**Published:** 2021-11-29

**Authors:** Juan Robles, Rubayat Qadeer, Tara Reyes Adames, Zoon Naqvi

**Affiliations:** ^1^ Department of Family and Social Medicine, Albert Einstein College of Medicine, Montefiore Medical Group, Bronx, New York, USA.; ^2^ Bronx Health Collective, Albert Einstein College of Medicine, Montefiore Medical Group, Bronx, New York, USA.

**Keywords:** mentorship, prehealth, socioeconomically disadvantaged, underrepresented minority

## Abstract

**Purpose:** Underrepresentation of racial and ethnic minorities in the health care workforce is a local and national issue. We describe and report on outcomes of a longitudinal service-driven prehealth pathway program in a low-income community intended to address this disparity and increase health equity.

**Methods:** The Bronx Community Health Leaders (BxCHL) is a prehealth pathway program for socioeconomically disadvantaged and underrepresented minority students seeking careers in health care. The program enrolls students in college or college graduates and provides longitudinal near-peer mentorship, exposure to the health care environment, and supports professional development. An academic federally qualified health center serves as the program's home site and learning environment. We conducted surveys and tracked the career advancement of program participants over a 6-year period, 2014–2020.

**Results:** One hundred sixty-eight students participated in BxCHL for >3 months. Of these, 76 students advanced into professional health career programs with 39 direct acceptances and 15 conditional acceptances to medical school programs, 9 nursing, 4 physician assistant, 9 health-related masters level programs, 1 respiratory therapy, and 1 optometry. The direct and overall acceptance (direct and conditional) rate of medical school applicants is 59% and 86%, respectively. The first 11 BxCHL alumni obtained their medical degree.

**Conclusions:** BxCHL's longitudinal service-driven and near-peer mentorship program design represents a replicable model to address health equity by supporting prehealth students from communities with limited access to mentors and professional learning environments in entering the health care workforce and serving their communities of origin.

## Introduction

Racial and ethnic minorities, including African Americans and Hispanics, comprise only about 6% of the US physician workforce, but comprise ∼30% of the US population.^1,2^ This disparity impacts the care of underserved populations, considering that one-third more underrepresented minority (URM) physicians practice in primary care settings compared with non-URM physicians.^3^ Also, greater diversity in health care has been associated with improvements in equity, increased access to care, better patient/provider communication, and overall satisfaction.^4^

Socioeconomically disadvantaged and URM students encounter many barriers and lack opportunities to enter the health care workforce.^5^ These barriers include lack of mentors and role models, and unfamiliarity with this career path.^6,7^ Prehealth pathway and enrichment programs continue to be key in recruiting, retaining, and advancing students by helping overcome these barriers and enhancing skills and competencies necessary to succeed in health care careers.^8^ The need for sustainable and longitudinal prehealth programs in low-income communities has taken increased urgency, particularly in light of the lack of black men in medicine.^9^

Several initiatives have contributed to advancing students in the Bronx community. For instance, the Bronx Health Opportunities Partnership-Einstein (Bronx-HOPE), a Health Careers Opportunity Program-funded program established by the Department of Family and Social at the Albert Einstein College of Medicine, sponsors prehealth summer enrichment programs.^10^ Similarly, Mentoring in Medicine, a community-based not-for-profit organization, facilitates student exposure to health care environments in the Bronx and Tri-state area.^11^ These programs offer mentorship and community service activities to spark students' interest and support persistence toward health care careers.^12,13^

Despite these and other national efforts, access to longitudinal mentorship networks and professional learning environments for URM prehealth students in low-income communities is severely limited.^7,14^ Most programs that offer these opportunities are longitudinally constrained by administrative costs and support for students.^15,16^

In response to the limited opportunities for exposure to medical environments and access to role models experienced by students in the Bronx community, Bronx Community Health Leaders (BxCHL) was created to serve as a longitudinal prehealth pathway program in a Federally Qualified Health Center (FQHC) setting sustained by service-driven student and characterized by near-peer mentorship to fill these training gaps and support needs. As a primary aim, we describe the program's design and report preliminary program and student outcomes. As a secondary aim, we examine the replicability and benefits of the longitudinal and flexible design of the BxCHL program.

## Methods

### Program design

The program was founded in 2014 by prehealth students serving as volunteers at a community health center. The students self-organized and developed a program characterized by near-peer mentorship, community service, and professional development. The program is primarily student-driven, sustained by the following: (1) four student “coordinators” with a 6-month nonpaid commitment to organize and schedule educational and community activities, and (2) teams of four to five members that sustain year-round educational and wellness programs for health center patients.

This design evolved out of need, as there was no site administrative support or resources to run the program. This design embraces the four pillars of successful longitudinal mentoring programs proposed by Mains and colleagues, including the ability to “*ignite the fire*,” “*illuminate the path*,” “*create the toolkit*,” *and* “*sustain the desire.*”^17^ The program's capacity is to support 50–60 students at any one time. Table 1 further outlines the program's design elements and activities.

**Table 1.** Program Design and Activities

Design element	Activities
Design pillars	Longitudinal near-peer mentorship Service-driven, student-driven Professional and leadership development Centered in the community
Target population	Socioeconomically disadvantaged and underrepresented minority students in college or graduated from college, age 18 and older
Recruitment and outreach	Program's website (bxchleaders.com), social media platforms Referrals from college advisors, providers at the health center, peer referral, and other prehealth programs in the community
Admissions	Online application, rolling and year-round Peer selection
Selection criteria	Demonstrates strong interest in community service Availability to participate in the program's weekly didactic activities No metric requirements
Membership expectations	Volunteer 2 h per week Attend 2 of 4 weekly meetings per month One scholarly presentation per year in a health-related topic Maintain up-to-date institutional identification Assume leadership roles
Organizational structure	4 program coordinators (6-month nonpaid commitment) 4 supporting leadership roles (e.g., community liaison, alumni liaison, scholarly work manager, coordinator trainer) 9 team leaders (4–5 students per team)
Meetings and frequency	Weekly 1-h coordinator meeting Weekly 1.5-h didactic meeting
Resources and cost and source of funding	Existing materials, equipment, and resources at the health center Overseen by a physician known as “program champion,” time protected about 0.1 full-time equivalent No administrative cost Year 1–3: no budget or funding Year 4–6: grant funding for direct support to students (∼$20,000/year) to participate in national or local scholarly forums, supplemental test prep courses, stipends for internship, and transportation
Program's motto	“It takes a village to raise a healthcare professional”
Community Service	Support patient care (e.g., patient navigation) and clerical tasks Conduct wellness programs (e.g., Yoga and dancing class, weight loss class) for patients Conduct educational programs (e.g., Help Desk, English class) for patients
Professional and leadership development	Attend presentations addressing social determinants of health or learn about health care professions from invited speakers Write project or poster proposals; attend and/or present at national or local scholarly forums Assume leadership roles such as coordinators, project point person, team leaders
Peer support and mentorship	Attend weekly meetings that allow for near-peer mentoring and networking; students are assigned a peer-buddy Receive mentorship and support from the program's alumni Students participate in team building activities
Career advising and support	Career advice and support completing the application to health professional programs including mock interviews

BxCHL enrolls students currently in college or college graduates in their “gap” year(s) before entering professional health career programs. Outreach occurs primarily through peer referral and community partners. Students submit an application that undergoes a peer selection process. It is a “rolling” admissions process year-round. The selection process seeks students with a strong commitment to community service and does not consider academic metrics. This selection process was agreed upon by the students. This approach aligns with health equity and holistic admission processes supported by other prehealth programs and medical school admission committees.^14,18^

Accepted students can remain in the program until they enter professional health career programs or can no longer regularly attend the program, but can re-enter the program at any point later in their prehealth journey.

The program's home site is the Montefiore Family Health Center (FHC), an academic family medicine FQHC and the training site for the Montefiore Medical Center/Albert Einstein College of Medicine Department of Family and Social Medicine Residency Program. The FHC serves a large immigrant, refugee, and underserved population in the Bronx. This location is easily accessible to the students by public transportation.

### Program activities

BxCHL focuses on applying evidence known to contribute to the success of socioeconomically disadvantaged and URM students pursuing health care careers. The program enhances competency-based skills identified by the American Association of Medical Colleges (AAMC) and most medical schools' holistic admission process, including interpersonal and communication skills, knowledge of health care systems, participation in scholarly inquiry, cultural competency, service orientation, and teamwork.^18,19^

In addition, exposure to near-peer mentors is known to contribute to the success of socioeconomically disadvantaged and URM students pursuing health care careers.^9,15^ Also, programs that expose students to health care environments have a positive impact on their decision to pursue health care careers.^20–22^

Table 1 highlights the program's main activities, Design pillars, Target population, Recruitment and outreach, Admissions, Selection criteria, Membership expectations, Organizational structure, Meetings and frequency, Resources and cost and source of funding, Program's motto, Community Service, Professional and leadership development, Peer support and mentorship, and Career advising and support. Its community service component is achieved through initiatives both at the health center and in the community. Students gain professional and leadership skills through participation in scholarly projects presented in various forums.

The program's near-peer mentorship component connects college, postbaccalaureate, and medical students. Students access mentors through a multidisciplinary and diverse health center and academic program staff. Students receive individualized support from program alumni, including writing personal statements, mock interviews, and letters of recommendation.

### Data collection

We conducted a survey (Qualtrics) over a 12-week period. Students were sent a survey link via e-mail, and participation was voluntary and confidential. An Institutional Review Board approval (IRB) (number 2019-10769) was issued by the Albert Einstein College of Medicine for this study. Using enrollment and attendance data, we identified all students who participated in the program for at least 3 months. We keep records of the students' application process to professional health career programs (cycles 2014–2020). We reached out via e-mail to students who had left the program to confirm their career progress.

We grouped students into three categories to examine the potential impact of length of participation in the program and their career advancement outcomes: (1) *active*—currently enrolled in the program, (2) *graduate*—student who persisted in the application process to enter health care professional programs while still in the program, (3) *associate*—students who transitioned through the program (were enrolled at least 3 months), and were not participating in the application process when they left the program.

## Results

A total of 194 students enrolled in the program from 2014 to 2020. There are 45 students identified as *active* members (currently enrolled in the program), 50 *graduates* (persisted in the program until advancement into the professional health career program), and 73 *associates* (participated in BxCHL and left the program before entering the professional health care programs). Of these, 168 (87%) remained in the program for at least 3 months. The survey response rate was 38% (64/168), with active and graduate students representing almost 90% of the respondents.

Table 2 shows the students' profile (*N*=64).

**Table 2.** Profile of Students Who Participated in the Program at Least 3 Months (2014–2020) (*N*=64)

Membership	Category^a^	Currently enrolled (%)	Graduate (%)	Associate (%)	Total (%)
	Enrollment status	45	50	73	168
	Survey respondents	31 (69)	26 (52)	7 (10)	64 (38)
Demographic profile when entered program					
Gender	Female	20 (65)	17 (65)	5 (71)	42 (66)
	Male	11 (35)	8 (31)	0	19 (30)
Member of the LGBTQ+ community		0	0	1 (14)	1 (2)
Age range	18–25	22 (71)	9 (35)	2 (29)	33 (52)
	26–30	8 (26)	14 (54)	3 (43)	25 (39)
	>30	1 (3)	2 (8)	1 (14)	4 (6)
Race/ethnicity	Black or African American	9 (29)	7 (27)	2 (29)	18 (28)
	Latinx	13 (42)	10 (38)	3 (43)	26 (41)
	Asian^b^	5 (16)	4 (15)	2 (29)	11 (17)
	White	1 (3)	3 (12)	0	4 (6)
	Multiple race/ethnicity	3 (10)	1 (4)	0	4 (6)
Financial/employment profile when entered program					
Household income	<$24,999	1 (3)	6 (23)	1 (14)	8 (13)
	$25,000–49,999	16 (52)	8 (31)	2 (29)	26 (41)
	$50,000–99,999	7 (23)	6 (23)	0	13 (20)
	>$100,000	1 (3)	2 (8)	0	3 (5)
	Don't know	2 (6)	0	0	2 (3)
Individual income	<$24,999	22 (71)	16	4	42 (66)
	$25,000–49,999	3 (10)	2 (8)	2 (29)	7 (11)
	$50,000–99,999	1 (3)	1 (4)	0	2 (3)
	>$100,000	0	0	0	0
	Don't know	2 (6)	0	0	2 (3)
Employment status	Full-time student, not working	3 (10)	6 (23)	0	9 (14)
	Full-time student, working part-time, <40 h/week	12 (39)	5 (19)	2 (29)	19 (30)
	Full-time student, working full-time, 40+ h/week	4 (13)	1 (4)	0	5 (8)
	Part-time student, working part-time, <40 h/week	1 (3)	5 (19)	0	6 (9)
	Part-time student, working full-time, 40+ h/week	3 (10)	0	1 (14)	4 (6)
	Not in school and not working	2 (6)	0	0	2 (3)
	Not in school, working part-time, <40 h/week	2 (6)	3 (12)	0	5 (8)
	Not in school, working full-time, 40+ h/week	3 (10)	4 (15)	3 (43)	10 (16)
	Not in school and not working—currently looking for work	1 (3)	0	0	1 (2)
	Other	0	1 (4)	0	1 (2)
Educational profile when entered program					
Highest degree attained or enrolled	Associate	1 (3)	0	0	1 (2)
	Bachelor's	26 (84)	23 (88)	4 (57)	53 (83)
	Master's	2 (6)	2 (8)	1 (14)	5 (8)
	Other	2 (6)	0	1 (14)	3 (5)
Type of college	Public city	18 (58)	7 (27)	3 (43)	28 (44)
	Public state	3 (10)	1 (4)	1 (14)	5 (8)
	Private liberal arts college (non-Ivy league)	9 (29)	11	2 (29)	22 (34)
	Ivy league	0	6	0	6 (9)
	Other	1 (3)	0	0	1 (2)
Science GPA	<3.0	6 (19)	7 (27)	1 (14)	14 (22)
	3.0–3.5	12 (39)	11	3 (43)	26 (40)
	>3.5	6 (19)	5	2 (29)	13 (20)
	I don't know	2 (6)	1 (4)	0	3 (5)
MCAT score (*N*=19)	<500	2 (11)	1 (5)	0	3 (16)
	500–505	4 (21)	1 (5)	2 (11)	7 (37)
	506–510	1 (5)	7 (27)	0	8 (42)
	>510	0	1 (5)	0	1 (5)
Prehealth journey and needs when entered program					
Intended health care professional program	MD	28 (90)	20 (77)	5 (71)	53 (83)
	Physician assistant	5 (16)	2 (8)	0	7 (11)
	Nursing	1 (3)	1 (4)	0	2 (3)
	Other health care careers	4 (13)	2 (8)	0	6 (9)
	Other nonhealth care careers	1 (3)	2 (8)	0	3 (5)
Gaps in education	*Took or plan to take gap year(s)*	28 (90)	22 (85)	6 (86)	56 (88)
Top reasons to take gap year (s)	*To do more health care-related activities*	21 (68)	16 (62)	2 (29)	39 (61)
	*To study for or retake MCAT*	19 (61)	13 (50)	4 (57)	36 (56)
	*To improve grades*	7 (23)	7 (27)	3 (43)	17 (27)
	*Needed to support family*	9 (29)	9 (35)	5 (71)	23 (36)
	*Needed to work to pay loans*	3 (10)	4 (15)	3 (43)	10 (16)
	*Was not sure of a career in health care and/or needed more time to decide*	3 (10)	2 (8)	0	5 (8)
	*“Was not sure if I could get into a professional program & needed more time to improve my overall academic & professional profile”*	14 (45)	13 (50)	3 (43)	30 (47)
	*Other reason(s)*	2 (6)	5 (19)	0	7 (11)
Prehealth activities before entered program	*Was part of any health care career interest group or premed club (*e.g., *MAPS)*	18 (58)	12 (46)	2 (29)	32 (50)
	*Shadowed health care professionals in your desired career*	15 (48)	18 (69)	6 (86)	39 (61)
	*Had a professional mentor or peer-mentor*	13 (42)	11 (42)	3 (43)	27 (42)
	*Volunteered in any health care setting (*e.g., *hospital, health center, nursing home)*	20 (65)	12 (46)	4 (57)	36 (56)
Motivations to enter BxCHL program	*I was looking for opportunities to volunteer and give back to my community.*	26 (84)	23 (88)	5 (71)	54 (84)
	*I was looking for opportunities to shadow and mentorship from health care professions.*	23 (74)	18 (69)	5 (71)	46 (72)
	*I was convinced by someone else.*	6 (19)	2 (8)	4 (57)	12 (19)
	*I was lost and lonely in the process to applying to health care careers and needed guidance and support.*	20 (65)	11 (42)	4 (57)	35 (55)
	*I needed to enhance my professional skills including interpersonal and communication skills.*	23 (74)	12 (46)	4 (57)	39 (61)
	*Other reason (s).*	1 (3)	2 (8)	1 (14)	4 (6)
Other educational and socioeconomically disadvantaged measures					
Educational barriers	*Comes from a low-income family*	24 (77)	16 (62)	4 (57)	44 (69)
	*First-generation college student*	19 (61)	15 (58)	4 (57)	38 (59)
	*Graduated from a high school where students had low average SAT/ACT scores or below the average state test result*	15 (48)	7 (27)	3 (43)	25 (39)
	*From a high school where at least 30% of enrolled students are eligible for free or reduced-price lunches*	16 (52)	11 (42)	4 (57)	31 (48)
	*From a school district where 50% or less of graduates go to college*	14 (45)	7 (27)	3 (43)	24 (38)
	*Does not consider English to be their primary language and for whom language is still a barrier to their academic performance*	8 (26)	4 (15)	2 (29)	14 (22)
	*Has a diagnosed physical or mental impairment that substantially limits participation in educational experiences*	4 (13)	1 (4)	0	5 (8)
	*Does not apply to me.*	0	5 (19)	0	5 (8)
Challenges faced in the last 12 months before COVID-19 pandemic	*Lack of transportation kept from attending school, work, or volunteer responsibilities*	10 (32)	2 (8)	0	12 (19)
	*Had to help with child care, an elderly, or sick adult in family*	16 (52)	9 (35)	4 (57)	29 (45)
	*Lack of support from family or other people during the most difficult times in career path*	17 (55)	4 (15)	3 (43)	24 (38)
	*Had to help family financially*	19 (61)	9 (35)	5 (71)	33 (52)

^a^ Definitions: *Graduate—*students who persisted and participated in the application process to enter professional health career programs while still in the program. *Associate—*students who transitioned through the program (were enrolled at least 3 months), and were not participating in the application process to enter professional health career programs when they left the program.

^b^ Mostly from Southeast Asia.

BxCHL, Bronx Community Health Leaders.

### Students' profile and barriers

Students are predominantly female (66%). The main age ranges are 18–25 (52%) and 26–30 (39%); they self-identified mainly as black/African American (28%), Latinx (41%), and Asian (17%). The group is primarily economically disadvantaged. The majority of students had work-related responsibilities while in school. At least 83% of the students had attained/or were enrolled in bachelor's degree when they entered the program. The majority attended a public city college (45%) and a private liberal arts college (non-Ivy league) (34%). Their intended career when they entered the program was mainly medicine (83%).

The students' prehealth journey is highlighted in Table 2**.** The majority indicated they took or were planning on taking “gap” years primarily for more exposure to health care-related activities and improving academic metrics. Less than half reported having a professional mentor or peer-mentor before entering the program, and a mentor was one of the top motivations for program enrollment. Of particular note, over half reported feeling “lost and lonely” in the process of applying to health care careers.

Notable socioeconomically disadvantaged measures in this group include growing up in a low-income family and being a first-generation college student. Other challenges faced in the last 12 months (before the COVID-19 pandemic) included the following: having to help their family financially (52%), help with child care or elderly/or sick adult in family (45%), lack of support from family or other people (38%), and at least 19% lacked transportation to attend school, work, or volunteer responsibilities.

### Impact

Combined, 76 students advanced into professional health career programs, with 39 direct and 15 conditional acceptances to medical programs, 9 nursing, 4 physician assistant, 9 masters level programs, 1 respiratory therapy, and 1 optometry (Table 3). The direct acceptance rate of medical school applicants is 59% and the overall acceptance (direct and conditional) is 86%. BxCHL students are accepted to competitive and top-tier medical school programs. The first 11 BxCHL alumni obtained their medical degrees.

**Table 3.** Health Care Professional Program Applicants and Matriculates by Application Cycles 2014–2015 Through 2019–2020

Application year cycle	Medical school applicants (graduate and associate)				Other health care professional programs	Total
	Applicant cohort (n)	Direct acceptance	Conditional acceptance^a^	Direct acceptance rate to USA MD/DO (overall)^b^		
2014–2015	1	1 (USA-MD)	—	100%	—	1
2015–2016	3	3 (USA-MD)	—	100%	—	3
2016–2017	13	8 (USA-MD) 1 (Caribbean)	2 (USAMD) 1 (USA-DO)	62% (92%)	1 (PA) 1 (Optometry) 1 (Nursing)	15
2017–2018	12	7 (USA-MD) 1 (USA-DO) 1 Caribbean)	1 (USAMD) 2 (USA-DO)	67% (92%)	3 (Nursing) 1 (MS)	16
2018–2019	12	2 (USA-MD) 1 (USA-DO) 2 (Caribbean)	4 (USA-MD) 3 (USA-DO)	25% (83%)	2 (PA) 1 (MS Nutrition) 5 (Nursing)	20
2019–2020	15	9 (USA-MD) 1 (USA-DO) 2 (Caribbean)	2 (USA-MD)	67% (80%)	1 (RT) 4 (MS) 1 (MS Nutrition) 1 (PA)	21
Total	56	39	15	59% (86%)	22	76

^a^ Includes postbaccalaureate programs that lead to medical school enrollment.

^b^ In parenthesis: Includes direct+conditional acceptance.

MS, master of science; PA, physician assistant; RT, respiratory therapist; USA-DO, US osteopathic school; USA-MD, US allopathic school.

Table 4 further highlights the students' outcomes by student categories and length of program participation. The students considered *associates* were in the program for a shorter period of time compared with the *graduates*. Of the students who left the program, many still remained on the prehealth path. Most students who were offered direct acceptance (21/27) and/or conditional acceptance (8/10) to medical programs were in the BxCHL program for more than 1 year.

**Table 4.** Career Advancement, Length of Program Participation by Student Category

Career advancement	Category			
	Currently enrolled (N=45)	Graduate (N=50)	Associate (N=73)	Combined (N=168)
Direct acceptance US allopathic school	—	25	5	30
Direct acceptance US osteopathic school	—	2	1	3
Conditional acceptance US allopathic school	—	6	2	8
Conditional acceptance US osteopathic school		5	2	7
Direct acceptance Caribbean medical school	—	2	4	6
Nursing	—	3	6	9
Physician assistant	—	1	3	4
Masters in sciences		3	2	5
Masters in nutrition	—	2	0	2
Other health care-related programs (e.g., optometry, respiratory therapy)	—	1	1	1
Still on prehealth path	44	—	20	64
Switched to non-prehealth path	1	—	5	6
Unable to contact	—	—	22	22
Length of program participation				
3–6 months	13	2	25	40
7–12 months	10	12	19	41
>1–2 years	14	20	13	47
>2 years	8	16	16	40

## Discussion

Since 2014, the BxCHL program has served as a support system and advanced many URM and socioeconomically disadvantaged prehealth students into professional health care programs. Medical school applicants have a direct accepted rate (59%) comparable or better than the national direct acceptance rate of 41.1% (white 44%, Latino 42%, African American 34%).^1^ These results are comparable or better than outcomes from similar pathway programs in advancing students to graduate programs and medical school acceptance.^15,23^ Eleven BxCHL alumni have obtained their medical degree and the majority will be training in primary residency programs. The BxCHL model was successfully replicated for another program.

The survey findings highlight the need for BxCHL-like programs to support URM and socioeconomically disadvantaged students in their prehealth path. While students face challenges that require significant societal intervention and resources, the BxCHL program addresses concrete needs such as providing funds for transportation and internships. It also addresses many students' sentiment of isolation (“lost and lonely”) during their journey. Students in these communities are at higher risk of not advancing due to the lack of social support or mentoring.

Data suggest that supporting these students would increase workforce diversity in health care professions and directly impact health care access and care of marginalized populations as they are more likely to practice in underserved areas.^24,25^

The data presented suggest that the longitudinal mentorship and flexible design of the BxCHL may increase the students' retention and success in their prehealth path, and some students may find a better occupational fit within the health care field. This longitudinal mentorship component is known to be important for the persistence and success of URM students in the prehealth path and has been recommended a critical element needed to address the national crisis of lack of black men in medicine.^9,26,27^

In general, the participation of students self-identifying as black or African American has ranged from 28% to 33% since BxCHL was established, which is higher than the current makeup of the physician workforce. Nonetheless, the ratio of female-to-male black or African American participants has been 3:1, which is similar to other prehealth programs in the community. This report also highlights challenges and opportunities in the delivery of prehealth programs in low-income communities. Notably, the program's attrition rate for the first 3 months of a student's participation in the program is about 13%.

However, we have addressed this by enhancing near-peer mentorship and striving for an inclusive and safe environment to reduce stereotype threats and imposter syndrome. This promotes camaraderie among students from marginalized communities and combats the feeling of being “lost and lonely,” increasing confidence and sense of belonging. The students are exposed to mentors from similar URM backgrounds who have traveled similar paths, allowing the students to envision themselves in health care careers, similar to those influences proposed for science, technology, engineering, and mathematics identity formation.^27^

The limitations in data include low student survey completion, which limited the ability to compare outcomes of the subgroups (*graduate* vs. *associate)* more robustly. Another limitation in this report is that it only includes self-reported academic metrics. Metrics is not a competency the program was designed to address directly, but might indirectly impact the students' academic performance considering that they are encouraged to maintain high academic standards as part of the program's expectations. Similarly designed programs with less rigid entrance metrics than traditional prehealth pathway programs have shown high success in advancing students into professional health care programs.^14^

An immediate goal is not only to optimize the program's design and activities but also to further understand its overall impact on students' personal and professional development. For example, to what extent does participation in the program affect the students' knowledge and attitude of professional health care careers? Could the program influence the students' career choice, including training in primary care and interest in caring for marginalized communities?

BxCHL has developed a student-driven, voluntarism-based organizational structure to sustain the program's activities year-round, and does not rely on administrative support. The program benefits from existing resources housed at the FQHC and is overseen by a physician “program champion,” who dedicates 10% of his time to this role. Grant funding has been used to enhance the program's activities and to support students. During the COVID-19 pandemic, the program successfully transitioned its activities to virtual learning platforms.

A BxCHL-like program called the South Bronx Community Health Leaders (SBxCHL) was successfully replicated at another intrainstitutional health center. A BxCHL replication guide and toolkit were produced and utilized as the framework for the new program. Several existing BxCHL students with experience in the program design and eager to replicate the sister program volunteered at the SBxCHL program pilot class. A family physician along with a global health fellow took on the role of program champions. SBxCHL has now been in existence for 2 years and currently supports 25 students.

Can and should health care and academic institutions commit to investing in programs such as BxCHL? Can these initiatives yield better outcomes in increasing diversity in the health care workforce than the existing efforts? Short term, the preliminary outcomes of the BxCHL program support this. Long term, we strongly believe that an initiative that produces homegrown health care professionals will address equity and improve health outcomes for underserved communities. This has already been on display as several BxCHL students have entered residency programs focusing on primary care.

BxCHL-like programs are more important than ever to help close the gap in educational opportunities and health disparities, especially during challenging times brought on by the COVID-19 pandemic.

To quote Emery CR and colleagues at the National Academy of Medicine, “To date, the majority of interventions to increase underrepresented minority participation in medicine have been undertaken within the existing systems and structures of academic medicine. To address the urgent need for a diverse physician workforce, we must use new and innovative tools to meet the current needs.”^28^ We could not agree more with this assessment; BxCHL's service-driven near-peer mentorship approach could be part of the solution. We envision a program model that can be replicated and made accessible to students in low-income communities to increase diversity in the health care workforce.

## Conclusions

We believe that the BxCHL program represents an innovative and viable program designed to recruit, retain, and advance prehealth students from communities with limited access to support systems and professional learning environments. It is a grassroots effort fueled by service-driven URM prehealth students that has resulted in tangible outcomes. Supporting BxCHL-like programs should be cost affordable for most health care and academic institutions. BxCHL programs are often the only support system for many students in this low-income community. Therefore, we call for the sponsorship and commitment of community and academic stakeholders. After all, this is consistent with the program's motto that “it takes a village to raise a healthcare professional.”

## Abbreviations Used

AAMC: American Association of Medical Colleges
Bronx-HOPE: Bronx Health Opportunities Partnership-Einstein
BxCHL: The Bronx Community Health Leaders
FHC: Family Health Center
FQHC: Federally Qualified Health Center
HRSA: Health Resources and Services Administration
IRB: institutional review board
SBxCHL: South Bronx Community Health Leaders
URM: underrepresented minority
